# Adaptive Feature Extraction for Blood Vessel Segmentation and Contrast Recalculation in Laser Speckle Contrast Imaging

**DOI:** 10.3390/mi13101788

**Published:** 2022-10-20

**Authors:** Eduardo Morales-Vargas, Juan Pablo Padilla-Martinez, Hayde Peregrina-Barreto, Wendy Argelia Garcia-Suastegui, Julio Cesar Ramirez-San-Juan

**Affiliations:** 1 Instituto Nacional de Astrofísica, Óptica y Electrónica, Luis Enrique Erro 1, Santa Maria Tonantzintla, San Andres Cholula 72840, Mexico; 2 Instituto de Ciencias, Benemérita Universidad Autónoma de Puebla, Puebla 72000, Mexico

**Keywords:** adaptive processing, mathematical morphology, laser speckle contrast imaging, blood vessel segmentation

## Abstract

Microvasculature analysis in biomedical images is essential in the medical area to evaluate diseases by extracting properties of blood vessels, such as relative blood flow or morphological measurements such as diameter. Given the advantages of Laser Speckle Contrast Imaging (LSCI), several studies have aimed to reduce inherent noise to distinguish between tissue and blood vessels at higher depths. These studies have shown that computing Contrast Images (CIs) with Analysis Windows (AWs) larger than standard sizes obtains better statistical estimators. The main issue is that larger samples combine pixels of microvasculature with tissue regions, reducing the spatial resolution of the CI. This work proposes using adaptive AWs of variable size and shape to calculate the features required to train a segmentation model that discriminates between blood vessels and tissue in LSCI. The obtained results show that it is possible to improve segmentation rates of blood vessels up to 45% in high depths (≈900 μm) by extracting features adaptively. The main contribution of this work is the experimentation with LSCI images under different depths and exposure times through adaptive processing methods, furthering the understanding the performance of the different approaches under these conditions. Results also suggest that it is possible to train a segmentation model to discriminate between pixels belonging to blood vessels and those belonging to tissue. Therefore, an adaptive feature extraction method may improve the quality of the features and thus increase the classification rates of blood vessels in LSCI.

## 1. Introduction

It is essential to visualize microvasculature within biological tissue because the visualization and location of blood vessels has several applications in the medical field; it can help to diagnose and treat illnesses such as retinopathy or port-wine stain, monitor perfusion around skin lesions, and assess the efficiency of photodynamic therapy, among others [1,2,3,4,5]. Furthermore, surgeons in the medical area can use measurements obtained from biomedical images as support in areas such as ophthalmology [1], dermatology [6], and neurosciences [7]. Measurements of blood vessels are essential to visualize the microvasculature and blood flow. Currently, researchers are developing techniques and computational algorithms to bring more precision to this task. Several methods for microvasculature visualization have been developed, but most of them are costly because of the required instrumentation and specialized medical machinery. The most common techniques are Doppler fluxometry, optical computer tomography, and magnetic resonance [7,8,9,10,11]. On the other hand, LSCI provides microvasculature visualization and high spatial resolution with simple instrumentation [12], capturing the movement of a sensed object by a blurring pattern formed in a Raw Speckle Image (RSI). The blurring pattern is studied through a CI to associate it with blood flow in tissue. Therefore, LSCI associates the blood flow velocity with a contrast value. A point-to-point analysis of the RSI is required to convert its grayscale values to contrast values. The process uses a squared analysis window to compute statistical values of a sample in the image, reducing the spatial resolution of the obtained CI. Loss of spatial resolution can be explained as a lack of contrast in the boundaries between blood vessels and tissue, making it difficult to determine some blood vessel characteristics, such as diameter. Current works on LSCI complement each other to fully implement the method in clinical practice. The main problems addressed are spatial resolution, high inherent noise in the CI, failure to satisfy ergodicity conditions, and the compromise between noise attenuation and temporal resolution [13,14,15]. Several methods have dealt with the loss of definition to improve the spatial resolution of the image, such as temporal, spatio-temporal, or spatial algorithms. Further, the window used affects the noise, since a square shape may not be ideal to compute a CI when blood vessels present different morphologies and sizes. Among the existing problems in LSCI, the high noise in the CI and the spatial resolution are addressed in this work.

The adaptive process of computing a contrast image aims to select a subset of pixels for each analyzed pixel in the RSI. It seeks to improve the contrast between regions in the CI by avoiding the combination of pixels between regions, obtaining more reliable statistics to improve the spatial resolution and obtain less-noisy images [16]. Thus, we hypothesized that using adaptive methods to compute features with pixels more representative of the analyzed area to feed a machine learning algorithm could improve the segmentation of blood vessels. The key contribution of this work is the analysis of adaptive feature extraction methods with simple statistics for blood vessel segmentation and contrast recalculation. Thus, it will improve blood vessel segmentation in LSCI and improve CI quality by reducing noise and improving spatial resolution. In addition, a method for adaptive processing of CI that considers the spatial relationship of pixels during adaptive structuring element retrieval is proposed. In this way, we aim to extend the capabilities of current adaptive processing methods to make them capable of improving CI even in small blood vessels.

## 2. Theoretical Framework

This section presents the theoretical framework required to understand the development of the proposed algorithms; it is divided into two major parts: laser speckle contrast imaging and image processing methods. The first part describes the methods to compute the contrast representation from the raw speckle images, divided into traditional, anisotropic, and adaptive. The second part introduces the state-of-the-art image processing techniques and evaluation metrics used in this work.

### 2.1. Laser Speckle Contrast Imaging

When a coherent light source, such as a laser, illuminates an object containing dynamic zones, the reflected and scattered light forms an oscillating pattern composed of bright and dark points. Thus, when a camera integrates the laser light while the illuminated region is moving, the speckles oscillate faster, creating a blurring pattern in the RSI. Therefore, the blurring pattern is associated with movement of an object, and a noisy pattern is related to a region with no movement. The study of the blurred pattern to locate and visualize blood vessels in the RSI is performed through a contrast representation ($K_{p}$) calculated using a sliding window *W* over the RSI by Equation (1) with values in the range [0,1]. The variables $\sigma^{W}$ and $\mu^{W}$ are the standard deviation and the mean, respectively, of the neighboring adjacent pixels of *p* in a squared neighborhood.

(1) $$K_{p}=\frac{\sigma^{W}}{\mu^{W}}$$

### 2.2. Traditional Methods in LSCI

In traditional methods, given a squared sample of pixels (*W*) around a pixel *p*, the contrast value represents how blurred the analyzed region is, and thus how much movement or speed is in the sensed tissue. When movement is found, the intensity values in $W_{p}$ are closer, and thus the obtained contrast value is near 0, which is more likely to represent a blood vessel. On the other hand, if no movement is found, the variance in $W_{p}$ is high, and therefore the contrast value is too; therefore, responses near to 1 are more related to tissue regions [12]. Among the most-used traditional methods, we can find the Spatial Contrast (sK) [12], Temporal Contrast (tK), and the Averaged Spatial Contrast (asK) [17,18]. sK processes one frame at a time by using a square analysis window of size d×d (Equation (2)). An analysis window of 5×5 is used to calculate CIs when the application requires more spatial resolution, and an analysis window of 7×7 is used when improved noise attenuation is required [19]. The main advantage of sK is the temporal resolution since one frame is required to obtain this CI. Nevertheless, when noise attenuation improvement is required (e.g., deeper blood vessels), asK provides the best compromise between noise and spatial resolution using 15 frames and a 5×5 window [18].

(2) $$sK_{x,y}=\frac{\sqrt{\sum_{i=x-r}^{x+r}\sum_{j=y-r}^{y+r}\frac{1}{d^{2}}{\left[RSI\left(i,j\right)-\mu_{x,y}^{s}\right]}^{2}}}{\mu_{x,y}^{s}}$$

where:

$$\mu_{x,y}^{s}=\sum_{i=x-r}^{x+r}\sum_{j=y-r}^{y+r}\frac{1}{d^{2}}RSI\left(i,j\right)$$

$$r=\frac{d-1}{2}$$

Finally, the tK defined by Equation (3) uses an anisotropic analysis window 1×n, analyzing one pixel along *n* frames. The tK method is noisier, but it also achieves the highest spatial resolution, preserving smaller blood vessels; to obtain a valid tK, at least 15 frames are necessary [17].

(3) $$tK_{x,y,n}=\frac{\sqrt{\sum_{f=1}^{n}\frac{1}{n}{\left[RSI\left(x,y,ri\right)-\mu_{x,y}^{t}\right]}^{2}}}{\mu_{x,y}^{t}}$$

$$\mu_{x,y,n}^{t}=\sum_{ri=1}^{n}\frac{1}{n}RSI\left(x,y,ri\right)$$

### 2.3. Adaptive Approaches in LSCI

Recent works suggest that applying a criterion to select the pixels involved in the contrast calculation can improve the quality of the CIs in LSCI [20,21,22]. The related adaptive processing methods are classified into three groups: those that select the size of *W* that best suits a blood vessel, methods that choose pixels following a specific direction, and methods that select pixels by comparing the central region of an AW with their connected pixels or similar methodologies.

The Multi Scale Contrast (msK) approach improved the quality of the CIs by selecting the window size d×d based on a granulometric analysis to match the size of the predominant blood vessel [22]. This approach obtained CIs with improved noise attenuation in general. Still, it reduces the contrast in the periphery of the blood vessels because of the combination of information by using a large analysis window. On the other hand, Three Sizes Contrast (TSK) computes the contrast representation by using squared AW with variable sizes of *W*, depending on the local variance of the analyzed pixel [23]. The method aims to compute the contrast representation using size d=3 in areas with less variance (dynamic region) and up to size 7 in areas with more significant variance (static region).

On the other hand, methods such as Anisotropic Contrast (aK) and Space Directional Contrast (sdK) compute contrast using a 1×n analysis window in the direction of the blood flow [20,21]. The aK estimates the direction of blood flow by minimizing the contrast in the analyzed pixel $p_{0}$ gradient within a set of windows in different directions *a*, obtaining CIs with noise attenuation. At the same time, it keeps an improved temporal resolution requiring only three frames for processing CI [20]. The sdK is similar to the aK because both perform adaptive computing by a directional analysis that maximizes the variance over a set *V* of directional windows $v_{i}$, performing an analysis of the RSI frame-by-frame, allowing better pixel selection to obtain improved CI by increasing the contrast between regions in the image and thus improving blood vessel visualization.

The main limitation of CI for microvasculature visualization in LSCI is the inherent noise present in the images, because current adaptive methods do not perform reliable pixel selection in noisy images. Thus, traditional adaptive methods for image processing cannot be used to perform pixel selection in an RSI to compute feature representation. Although current anisotropic and adaptive methods increase the contrast between regions, they introduce artifacts in the resulting CI derived from the 1×n pixel selection. Therefore, there is still room for improvement in adaptive methods.

### 2.4. Jaccard Index

The Jaccard Index (JI) is a similarity coefficient between two sets *A* and *B*. In the image processing area, JI can be used as a segmentation overlap coefficient between two binary images to determine the segmentation quality defined in Equation (4). Is is the segmented image, and Igt is related to the ground-truth image, TP are the true positives, TN are the true negatives, and FN are the false negatives.

(4) $$JI\left(Is,Igt\right)=\frac{Is\cap Igt}{Is\cup Igt}=\frac{TP}{TP+FP+FN}$$

### 2.5. Image Normalization

Image normalization is used to stretch the range of intensity values by scaling them into a new range $\left[g_{max},g_{min}\right]$ given by a max value $g_{max}$ and a min value $g_{min}$ [24]:

(5) $$I_{n}\left(x,y\right)=\frac{g_{max}-g_{min}}{I_{max}-I_{min}}\left(I\left(x,y\right)-I_{min}\right)+g_{min}$$

where $I_{n}$ represents a normalized pixel of image *I*, and $I_{max}$ and $I_{min}$ are the maximum and minimum values, respectively.

### 2.6. Image Segmentation

In computer sciences, assigning a class label using computational models to an observation of features extracted from an entity is known as classification. Classification models often use statistical representation from the data, known as features. When the classification task is performed pixel-by-pixel in an image, it is called segmentation, which refers to the process of assigning a label to each pixel in it. There are two types of classification: when the label for each data observation is known and the process is guided, it is called supervised learning; otherwise it is called unsupervised learning.

Given a set of *n* data points, also called observations. The goal of classification is to infer an unknown relation between the features $f_{i}$ and a class $c_{i}$. More formally, the process is defined as c=m(f) such that m(f):F→C [25]. The observations are of the form $D=\{<f_{1},c_{1}>,<f_{2},c_{2}>,\ldots <f_{n},c_{3}>=<F,C>\}$, where *f* can be a vector with a set of features $f_{i}=\left\{f_{1},f_{2},\ldots f_{n}\right\}$, and $c_{i}$ is the class label. The features are usually descriptors of the observations, i.e., statistical measures from a set of pixels of an analysis window. When the analysis window is squared $W^{d}$ with size *d*, we call it traditional feature extraction, and when the pixels used for feature extraction are a $\subseteq W^{d}$, then we call it adaptive feature extraction. On the contrary, unsupervised learning is a technique that consists of grouping data according to a given criterion without using any class label guiding the training of the algorithm.

## 3. Materials and Methods

This section presents the experimental setup used to acquire the images for the experimentation and the proposed methodology used to perform the adaptive feature extraction and contrast recalculation.

### 3.1. Experimental Setup for Image Acquisition

**Figure 1:**
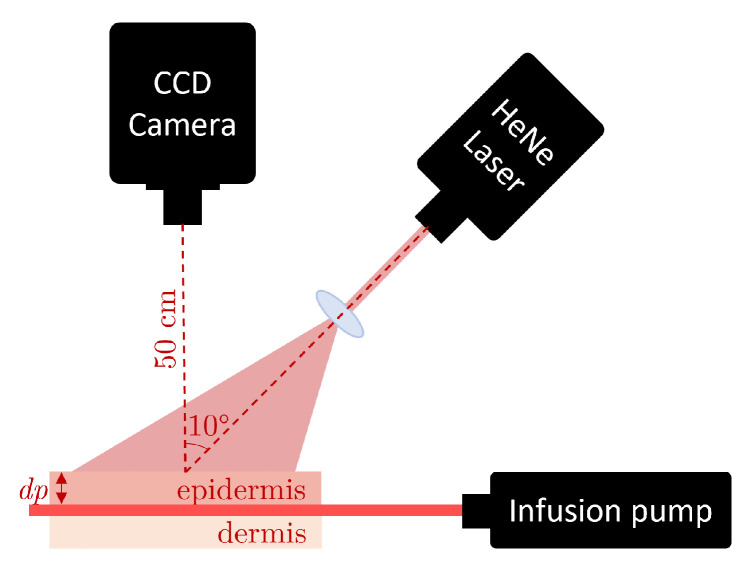
Experimental setup used to acquire the RSIs in vitro samples.

A CCD camera (Retiga 2000R, Qimaging, Tucson, AZ, USA) equipped with a zoom lens (NAV ITAR ZOOM 700) was employed to capture the RSIs with magnification = 1, and different exposure times and 2 pixels/speckle for in vitro and in vivo datasets [26,27]. A polarizing filter was mounted in front of the zoom lens and perpendicularly oriented to the polarization of the incident light (He–Ne laser at 632.8 nm, 15 mW) to mitigate specular reflectance from the samples. The in vitro images were obtained from skin phantoms. We constructed skin phantoms using an epoxy resin block with appropriate concentrations of TiO${}_{2}$ powder (TiO${}_{2}$ 1.45 mg/mL) to simulate the optical properties of the dermis. Later, a capillary glass tube with an inner diameter of 700 μm was embedded at the surface of the block (thinXXS Microtechnology AG, Zweibrücken, Germany). We placed thin silicone phantoms (polydimethylsiloxane TiO${}_{2}$ powder 2 mg/mL) of varying thicknesses (190 to 1000 μm) on top of the resin block to simulate the epidermis. An infusion pump (Model NE-500, New Era Pump System Inc., Farmingdale, NY, USA) was used to simulate the blood flow passing by a solution (1% concentration) into a microchannel via Tygon tubing. The complete experimental setup can be observed in Figure 1.

**Figure 2:**
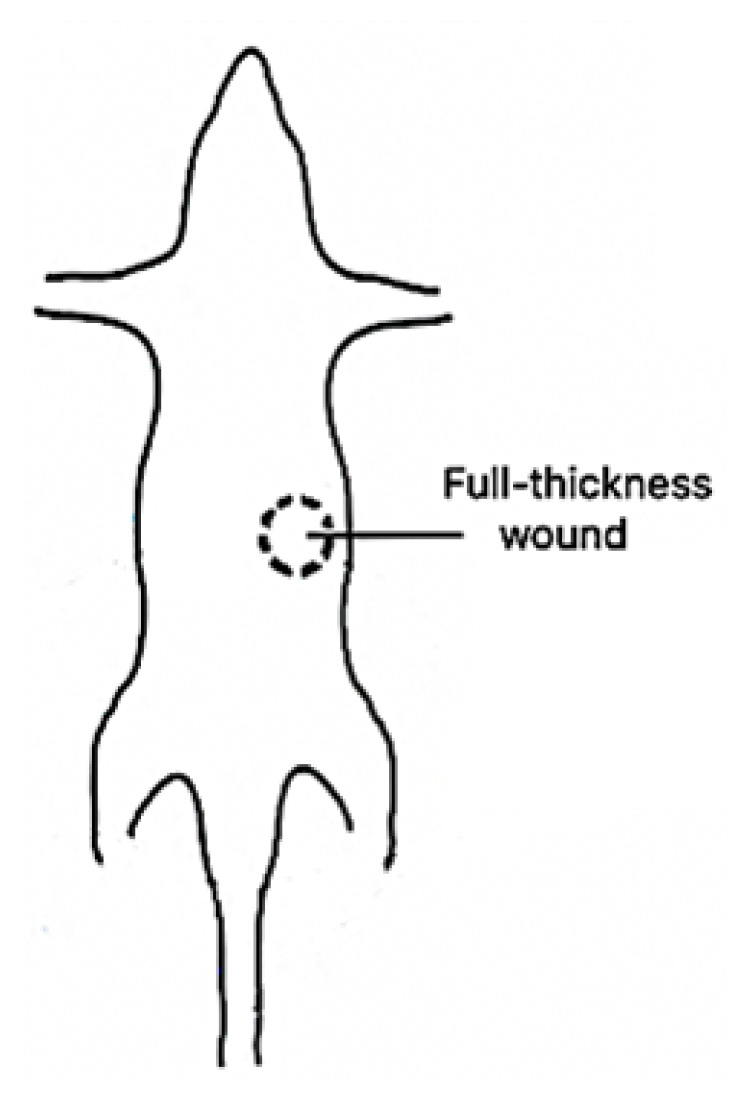
Surgical procedure to acquire the in vivo speckle images.

One male rat (Rattus norvegicus) weighing 120 g was anesthetized intraperitoneally with xylazine and ketamine hydrochloride in doses of 0.1 and 0.7 mg/100 g body weight, respectively, for the in vivo experiments. A circular template 1 cm in diameter was used to outline the wound size on the dorsal skin of the rat (see Figure 2). Later, a full-thickness wound corresponding to the template area was created using sterile surgical scissors and forceps. It is important to mention that the wound was made down to the fascial layer to expose the blood vessels. All animal procedures were performed in accordance with the Mexican Norm NOM-062-ZOO-1999, and the experimental protocol (GASW-UALVIEP-17) was approved by the Internal Committee for Care and Use of Laboratory Animals (CICUAL) at Benemérita Universidad Autónoma de Puebla (BUAP). To acquire the speckle images, the wound was sandwiched between two aluminum plates with a perforation 1.1 cm in diameter. The area of the wound coincided with the perforation area of the plates so that a beam laser (633 nm wavelength) passed through the wound, and the blood vessels were projected on a CCD. A saline solution was applied periodically in the subdermal layer to prevent dehydration.

### 3.2. Adaptive Feature Extraction in LSCI

This work is focused on the classification improvement of blood vessels at different depths. In general terms, the method performs a punctual analysis pixel-by-pixel, calculating a binary mask with two possible values indicating whether a pixel is used or not to operate with a feature extraction method δ to compute the set of features $f=\{\delta_{1},\delta_{2},\ldots ,\delta_{n}\}$, where δ is a feature extraction operation such a texture or statistical analysis.

The solution to segment blood vessels in LSCI by calculating the features using adaptive processing starts by computing a reference image to identify the pixels used to calculate the features. The solution is divided into three steps: (i) reference image retrieval, (ii) feature extraction, and (iii) classification. The first stage computes a reference image *R* to perform pixel selection. *R* is needed to determine whether a pixel belongs to a certain distribution to avoid noisy and outlier values in the RSI. First, a CI *k* is obtained. Later, the CI obtained from the RSI *I* is grouped into clusters to avoid outliers in the selected values. Then, a mask $S_{x}^{d}$ is calculated for each pixel in *I* to compute the features adaptively. Therefore, the mask is calculated using a region-growing process using the currently analyzed pixel *x* as a seed. The region-growing process is performed over a traditional analysis window superimposed in *x* with a size of *d*, and it selects the pixels that will be operated with the criterion of Equation (6). Later, the feature set is calculated with the superimposed *W* pixels marked as 1 in the mask $S_{x}^{d}$. Finally, a classification model is trained with the feature representation *F* in the third step.

The Adaptive window Contrast (awK) [28] and the proposed Spatially Adaptive Windowing Contrast (sawK) derived from the awK were used to perform the experiments. The awK uses the k-NN algorithm to perform the grouping phase. In this manner, the analysis matrix is formed by reducing the intra-class variance by using Equation (6) as criteria.

(6) $$S_{p}^{d}\left(p_{i}\right)=\left\{\begin{array}{cc} 1, & R\left(p_{o}\right)=R\left(p_{i}\right)\\ 0, & \mathrm{otherwise} \end{array}\right.$$

The awK may combine information from the dynamic and static region in the smallest blood vessels or where the blood flow is insufficient, attacking the problem; we opted to include spatial distance in the grouping of the reference image to perform pixel selection. On the other hand, the sawK uses the Sequential Linear Iterative Cluster algorithm to evaluate and generate pixel groups by using contrast intensity and spatial distance as a similarity distance [29].

A postprocessing step selects the appropriate pixels, avoiding a posterizing effect when the features are calculated. A set of *k* centers are initialized in regular intervals around the image. The distribution is defined by $L=\sqrt{N/k}$ in *R*, where *N* is the number of pixels in the input image. First, the centers are moved to the lower gradient in a 3×3 neighborhood. This step is performed to avoid allocating a center in the periphery of the blood vessel and outlier values. Next in the assignment step, a label l(p)=−1 is assigned for each pixel *p*. Then, the pixels at a maximum distance of 2L from each center and adjacent pixels are labeled. Later, each cluster is updated until they no longer change. Then a pixel-by-pixel analysis is performed. A distance that combines the contrast value and the proximity into a single measure is used to determine whether the pixel is included in the calculation or not (Equation (7)).

(7) $$D=ds+\frac{M}{S}dc$$

where:

$$ds=sqrt{\left(R_{i,j}-R_{x,y}\right)}^{2}$$

$$dc=sqrt\left[{\left(i-x\right)}^{2}+{\left(j-y\right)}^{2}\right]$$

**Figure 3:**
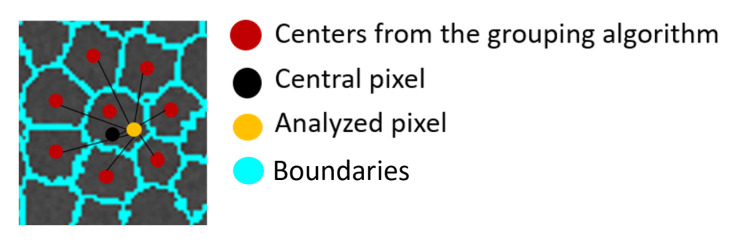
Adjacency of the analyzed pixels with the cluster centers.

The first step of pixel selection consists of calculating a distance between the analyzed pixel pp, the central pixel *p*, and the centers obtained from a grouping algorithm that considers the spatial distance to perform the clustering process. If the minimum distance from the three distances is to *p*, then pp is set as 1 in $S_{p}^{d}$ (Figure 3). Then, the contrast is calculated as the awK.

## 4. Experiments and Results

The images used in the experiments consist of three sets: two sets are of simulated blood vessels and one is in vivo. The first one consists of straight and bifurcated blood vessels with fixed mote size, exposure time, and flow with variable depth. The second set consists of blood vessels with fixed mote size and flow and variable depth and exposure time. Further, a set of simulated straight and bifurcated blood vessels are used to test the factors that affect the segmentation of blood vessels by using adaptive methods. On the other hand, a set of in vivo images is used for validation in a more realistic environment. The experiments test the hypothesis that adaptive approaches for feature extraction can improve segmentation of blood vessels. Most of the presented experiments focus on testing the parameters influencing the results. The JI was used as an evaluation metric in the periphery of the blood vessels to avoid bias in the measurements because we focus on improving spatial resolution. The results are grouped into adaptive (awK and proposed sawK) and a classical squared AW with size d=5. The evaluation of segmentation and classification algorithms is not intended in this work. For this purpose, a k-NN model was trained with statistical features such as the sum, max and min values, mean, range, standard deviation, and entropy with 10-fold cross-validation for replication.

**Figure 4:**
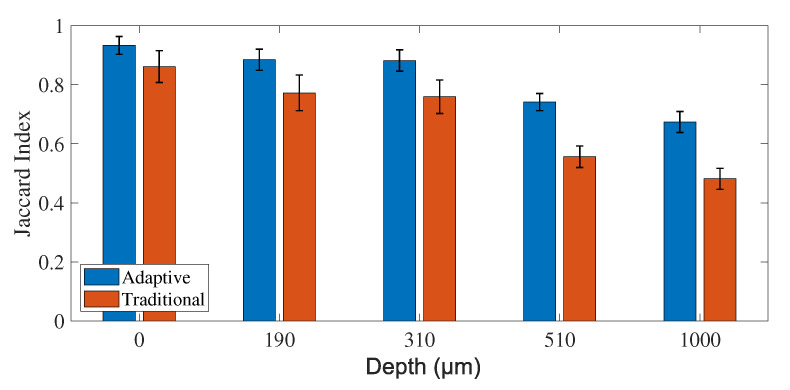
Comparison of JI obtained with traditional (red) and adaptive feature extraction (blue), performing the classification with a weighted k-NN. Results are grouped by depth to study depth invariance. ANOVA (F-Value = 2413.46, *p* = 0, α = 0.05).

The first experiment consists of the analysis of exposure time to determine the temporal resolution of the model. The exposure time determines the intensity of the blurring pattern by increasing or decreasing it as a function of the seconds that the camera senses information. If it is lower than the slowest fluctuation in the speckle pattern, the blurring is insufficient to detect movement in the image. On the other hand, if the exposure time is very long, the static pattern could be averaged and confused with blood vessels. The controlled factors in these experiments are depth and exposure time, where dp={0, 190, 310, 510, and 1000} μm, and et={70, 138, 256, 500, 980, 1883, 3949, 5908, 8204, 11,062, 12,200, 20,885, 26,481, 31760, and 32,789} μs, respectively. In order to obtain different exposure times, we use a set of neutral density filters (NDF) to compensate for the light intensity on the CCD camera. For a specific NDF, automatic estimation of the exposure time is performed via software. The results suggest that it is possible to improve the segmentation results for all the depths independent of the exposure time, with more significant results at the profound depths (Figure 4).

**Figure 5:**
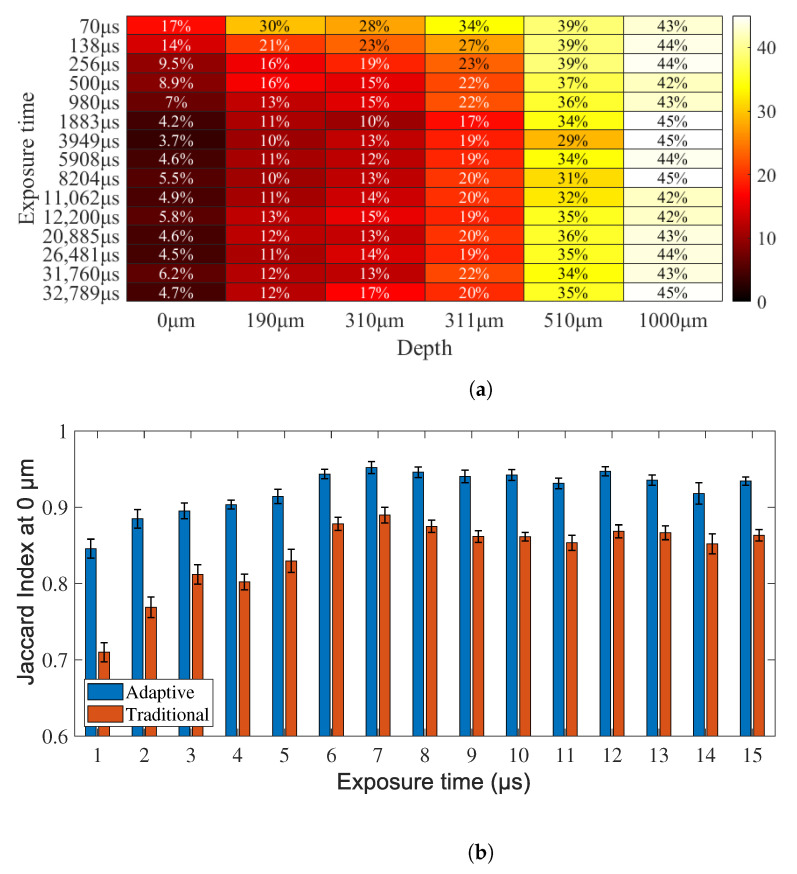
Comparison of traditional (red) and adaptive feature extraction (blue), performing the classification with a weighted k-NN with all the exposure times and depths. (**a**) shows the differences between the adaptive traditional methods in terms of percentage improvements, and (**b**) shows the results grouped by exposure time at 0 μm. ANOVA (F-Value = 22.43, *p* = 0, α = 0.05).

Figure 5a presents the results contrasting the exposure time with the depth in terms of improvement percentage and shows a more significant increase in the longest exposure times, stabilizing at exposure times higher than 980 μs. It can be inferred that depth has more of an impact on image quality than exposure times, increasing it from 4.7% to 17% in the shallow depths and 43% to 45% at profound depths (1000 μm).

**Figure 6:**
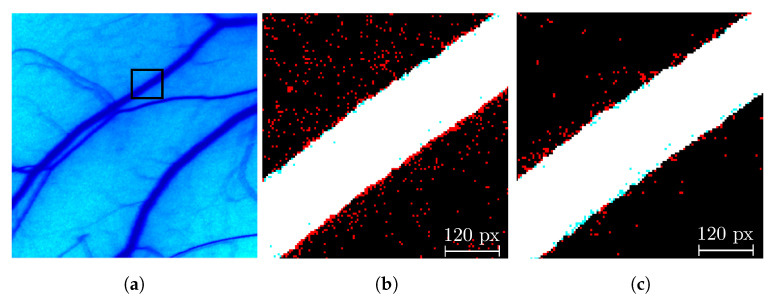
Segmentation results: (**a**) CI of an in vivo image of the used data where the square is the close-up analyzed in (**b**,**c**); (**b**) is the traditional segmentation result, and (**c**) is the adaptive segmentation results. Pixels in black represent true negatives, white are true positives, red are false positives, and blue are false negatives.

A representative patch from the in vivo dataset is shown in Figure 6 for comparison. Figure 6b represents a segmented patch with the traditional method, and Figure 6c is a segmented patch with the adaptive method. There are two main improvements in the segmentation that can explain the JI increase. The noise reduction and the improved statistics dismissed internal and external blobs (red dots and blue blobs, respectively). On the other hand, the improved spatial resolution derived from the pixel selection leads to better segmentation in the periphery of the blood vessels.

**Figure 7:**
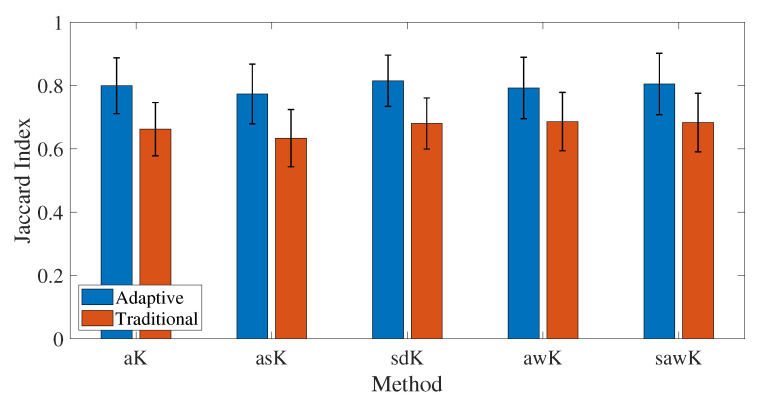
Comparison of the segmentation results by method for the analyzed depths and variable exposure times: Anisotropic Contrast (aK), Averaged Spatial Contrast (asK), Space Directional Contrast (sdK), Adaptive window Contrast (awK), Spatially Adaptive Windowing Contrast (sawK). ANOVA (F-Value = 27.70, *p* = 0, α = 0.05).

Finally, an experiment is performed to establish the independence of the feature extraction method to the contrast representation used to extract the features required to train the classification algorithm. The aK, asK, sdK, awK, and sawK methods are used to extract the features to know the effect of the method on the JI. The features are later used to train the classification models. Adaptive feature extraction obtains a higher JI independent of the method used, as seen in Figure 7, which means improvement in the segmentation of blood vessels in LSCI. Therefore, adaptive feature extraction may be invariable to the CI used. The results are invariant for each method, but the JI increases for all of them.

### 4.1. Contrast Recalculation

Although the segmentation of blood vessels in LSCI obtained significant results with adaptive processing, the segmentation can also be used to perform a contrast recalculation by using the same method for feature extraction, calculating only the mean and standard deviation to generate a CI. First, a pre-processing step is required to eliminate all the isolated pixels and blood vessels with holes to use the segmented image as a reference to perform the pixel selection. If the segmented image is used, the isolated pixels can lead to a selection of AW of size 1×1, obtaining a sample of one pixel, and thus, the statistics cannot be calculated.

**Figure 8:**
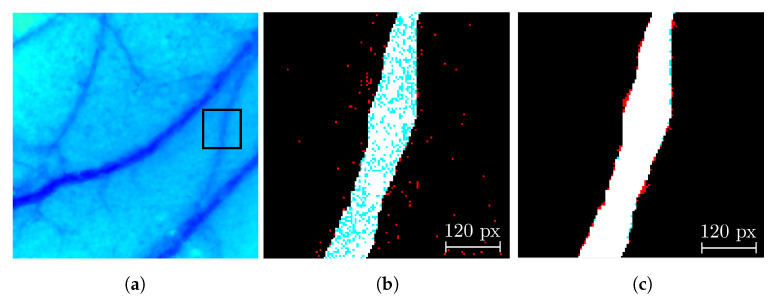
Comparison of the segmentation results before and after the reconnection process: (**a**) CI of an in vivo sample where the square represents a close-up of the (**b**) output of the segmentation algorithm and (**c**) the reconnected blood vessel. Pixels in black are true negatives, white are true positives, red are false positives, and blue are false negatives.

First, a morphological closing with a structural element of type disk eliminates all those isolated pixels in the background region while maintaining the inner area of the Region Of Interest (ROI) with minor changes. If pixels in the inner region of the blood vessels are dismissed, then reconnection of the regions cannot be performed. A radius of 1 for the structural element avoids thickening the periphery of the blood vessel and thus reduces the spatial resolution. Later, a morphological area filter eliminates all the objects with less than 25 pixels in the background region of the image to avoid the contrast calculation with fewer pixels than those in a 5×5 analysis window. Finally, a reconnection step analyzes the inner region of all the structures in the image. Based on its surroundings, a label is assigned to all those isolated structures based on their neighborhood. The neighborhood is analyzed to determine if a tissue pixel is labeled as a blood vessel. If there is at least one pixel in each direction for four connectivity adjacencies around it, the pixel is marked as a blood vessel. Otherwise, its label remains unchanged. Then, with this process, the isolated pixels are eliminated outside the blood vessels, and the inner region is connected [16], as seen in Figure 8, which shows a decrease of the FP due to the morphological closing (reduction of red blobs) and a reduction of the FN in the inner region of the blood vessels or wrongly classified pixels as tissue seen as blue blobs inside the blood vessels.

**Figure 9:**
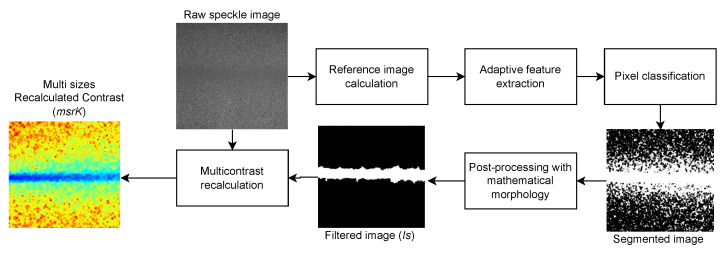
Methodology for the contrast recalculation using adaptively extracted features.

The noise reduction and elimination of disconnected pixels allows the use of the segmented image as a reference to recalculate the contrast, as awK does, by using Equation (6), the process of which is depicted in Figure 9.

**Figure 10:**
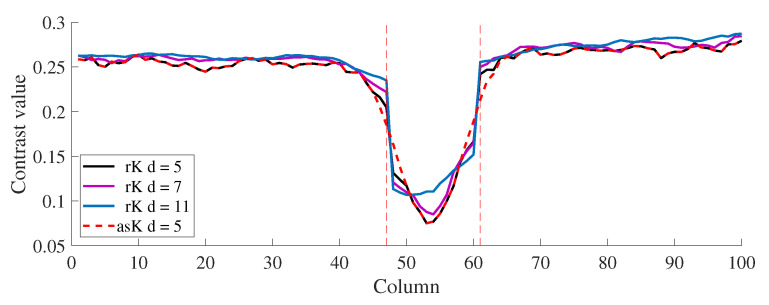
Slice of a contrast image for contrast image calculated using a traditional (red) and an adaptive approach (blue) for an in vivo image.

Slices taken from representative regions of the recalculated images are shown in Figure 10, in which the vertical dotted line indicates the periphery of the visualized blood vessel. As seen in Figure 10, contrast recalculation can increase the definition of the blood vessels in its periphery with the drawback of increasing the contrast values at its center, the effect of which increases along with the size of the maximum analysis window.

**Figure 11:**
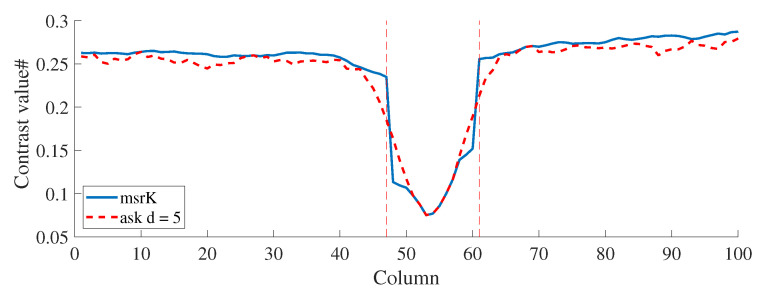
Slice of a CI calculated using a traditional analysis window with d=5 and by using the multi-scale contrast with the recalculated contrast.

Although the main contributions of the recalculated contrast are the improved noise attenuation and spatial resolution, improving the quality and visualization of blood vessels, a postprocessing step can be performed to combine the best of asK and the recalculated contrast. In order to maintain the minimum value of contrast at the center of the blood vessel and the improved spatial resolution and noise reduction in the outer region, Equation (8) can be used at each pixel to obtain a Multi-Scale Recalculated Contrast (msrK) image (Figure 11). This can be useful in medical applications such as the measurement of the diameter of a blood vessel or assessment of diabetic foot ulcer healing [30,31].

(8) $$msrK\left(x,y\right)=\left\{\begin{array}{cc} min(rK^{5},rK^{7},\ldots rK^{d}) & I_{s}\left(x,y\right)==0\\ max(rK^{5},rK^{7},\ldots rK^{d}) & otherwise \end{array}\right.$$

### 4.2. Establishing the Differences between awK and sawK

**Figure 12:**
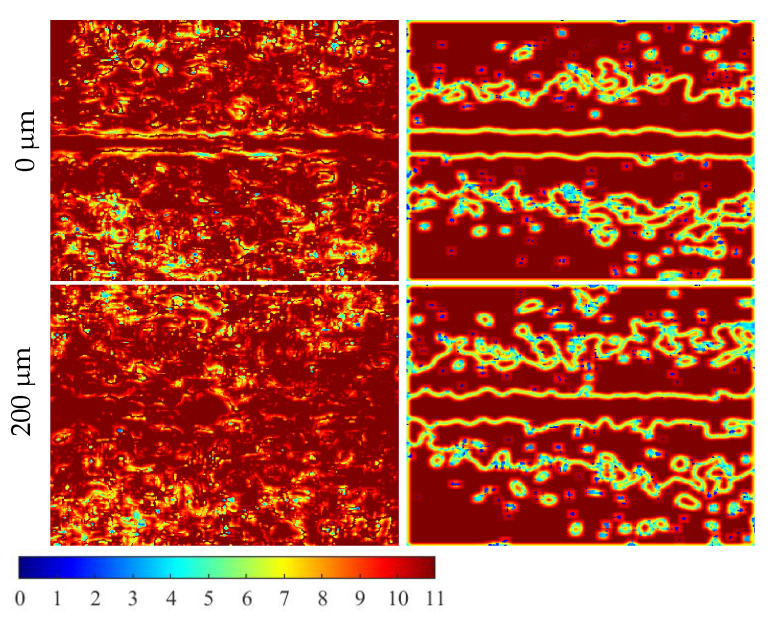
Comparison of maps sizes for the adaptive processing of LSCI of an in vitro CI with size of 344×329 pixels. The depth of the blood vessel and the adaptive method vary.

To compare the awK and sawK, an image showing the number of pixels required to compute the contrast at each pixel in the glsRSI is obtained. Zp=|Spd| (Figure 12) is the representation, commonly known as a size map. With the exception of the perimeter of the blood vessels and transitional regions, the awK and sawK use an analysis window of 11×11 in practically all of the images. The distinction between the awK and the sawK is in the identification of the ROIs, as seen in Figure 12. Meanwhile, the analysis window size in awK is determined by the correct amount of clusters: the blood vessel, the transition region, and the static region. Blood vessels with different contrast values are hence the awK’s limiting factor. The spacing between regions may not be adequate if the contrast value between blood vessels is too small, the main limitation of sawK being the parameter selection (separation between regions and spatial distance influence).

## 5. Conclusions

The main limitation in the segmentation of blood vessels in LSCI is the intense noise inherent to the CIs. Although several methods increase the quality of the images, state-of the-art methods introduce artifacts in the CI, which leads to a reduction in the segmentation rates. Thus, this work presents an analysis to know if the proposed method sawK and the adaptive awK can improve the classification of blood vessels derived from the improved noise attenuation, spatial resolution, and image quality after a contrast recalculation process.

The sawK and awK use the advantages of unsupervised learning to select the pixels involved in contrast calculation with anticipation. The use of spatial distance is essential in cases where the contrast value is insufficient to discriminate between the static region and blood vessels. Therefore, the grouping methods allow adaptive contrast calculation to select pixels, avoiding the limits between regions and resulting in less noisy features with improved spatial resolution. Thus, classification rates are improved when false positives in the outer region of the blood vessel and false negatives in its periphery are reduced.

The adaptive feature extraction method can improve the classification rates significantly under several conditions. It can be used to locate blood vessels independent of the contrast method used as a feature, the camera’s exposure time, and the blood vessel’s depth and morphology compared with traditional feature extraction, which uses square analysis windows. The adaptive feature extraction method can reduce the false positives in the classification by reducing the outlier values in the outer region of the blood vessels, which means more stable or smoothed features. On the other hand, the increase in spatial resolution shows a reduction in the false negatives in the periphery of the blood vessel. Further, the increased spatial resolution can lead to better diameter estimation of blood vessels for medical applications.

On the other hand, the segmented markers can be used as a reference image to adaptively recalculate the contrast representation after a postprocessing step, eliminating the outlier values and improving the disconnected blood vessels. The main drawback is a reduction of the contrast value at the center of the blood vessels compared with a traditional contrast with an analysis window of size 5×5. To address the problem, we selected an appropriate contrast value using TSK. Then, the results suggested that the combination of traditional approaches and the proposed recalculation of contrast maintains the blood flow values in the center of the blood vessels, reduces noise, and maintains increased spatial resolution. Finally, although the proposed methods showed good performance in the domain, further analysis could be required to use the techniques in images with several objects.
